# Bovine cryptosporidiosis: impact, host-parasite interaction and control strategies

**DOI:** 10.1186/s13567-017-0447-0

**Published:** 2017-08-11

**Authors:** Sarah Thomson, Carly A. Hamilton, Jayne C. Hope, Frank Katzer, Neil A. Mabbott, Liam J. Morrison, Elisabeth A. Innes

**Affiliations:** 1 Moredun Research Institute, Pentlands Science Park, Bush Loan, Edinburgh, EH26 0PZ Scotland, UK; 2 The Roslin Institute & Royal (Dick) School of Veterinary Sciences, University of Edinburgh, Easter Bush, Midlothian, EH25 9RG UK

## Abstract

Gastrointestinal disease caused by the apicomplexan parasite *Cryptosporidium parvum* is one of the most important diseases of young ruminant livestock, particularly neonatal calves. Infected animals may suffer from profuse watery diarrhoea, dehydration and in severe cases death can occur. At present, effective therapeutic and preventative measures are not available and a better understanding of the host–pathogen interactions is required. *Cryptosporidium parvum* is also an important zoonotic pathogen causing severe disease in people, with young children being particularly vulnerable. Our knowledge of the immune responses induced by *Cryptosporidium* parasites in clinically relevant hosts is very limited. This review discusses the impact of bovine cryptosporidiosis and describes how a thorough understanding of the host–pathogen interactions may help to identify novel prevention and control strategies.

## Introduction

*Cryptosporidium parvum* was first described in 1907 by Edward Ernst Tyzzer in the small intestine of mice [1]. Since then, over 30 species of *Cryptosporidium* have been described that infect a wide range of host species [2]. Several species infect cattle and have a significant impact upon animal health and production, especially in young calves. Unfortunately, relatively few tools are available to combat bovine cryptosporidiosis (no vaccine and one drug of limited utility), and our knowledge of host–pathogen interactions in the bovine host is also very limited. Addressing these important gaps in our understanding of bovine cryptosporidiosis will aid the development of interventions going forward. This review summarises our current understanding of bovine cryptosporidiosis, with a particular focus on what is currently known about the bovine immune response to this pathogen, and discusses avenues for new research to further our understanding of host-parasite interactions in bovine cryptosporidiosis.

**Figure 1:**
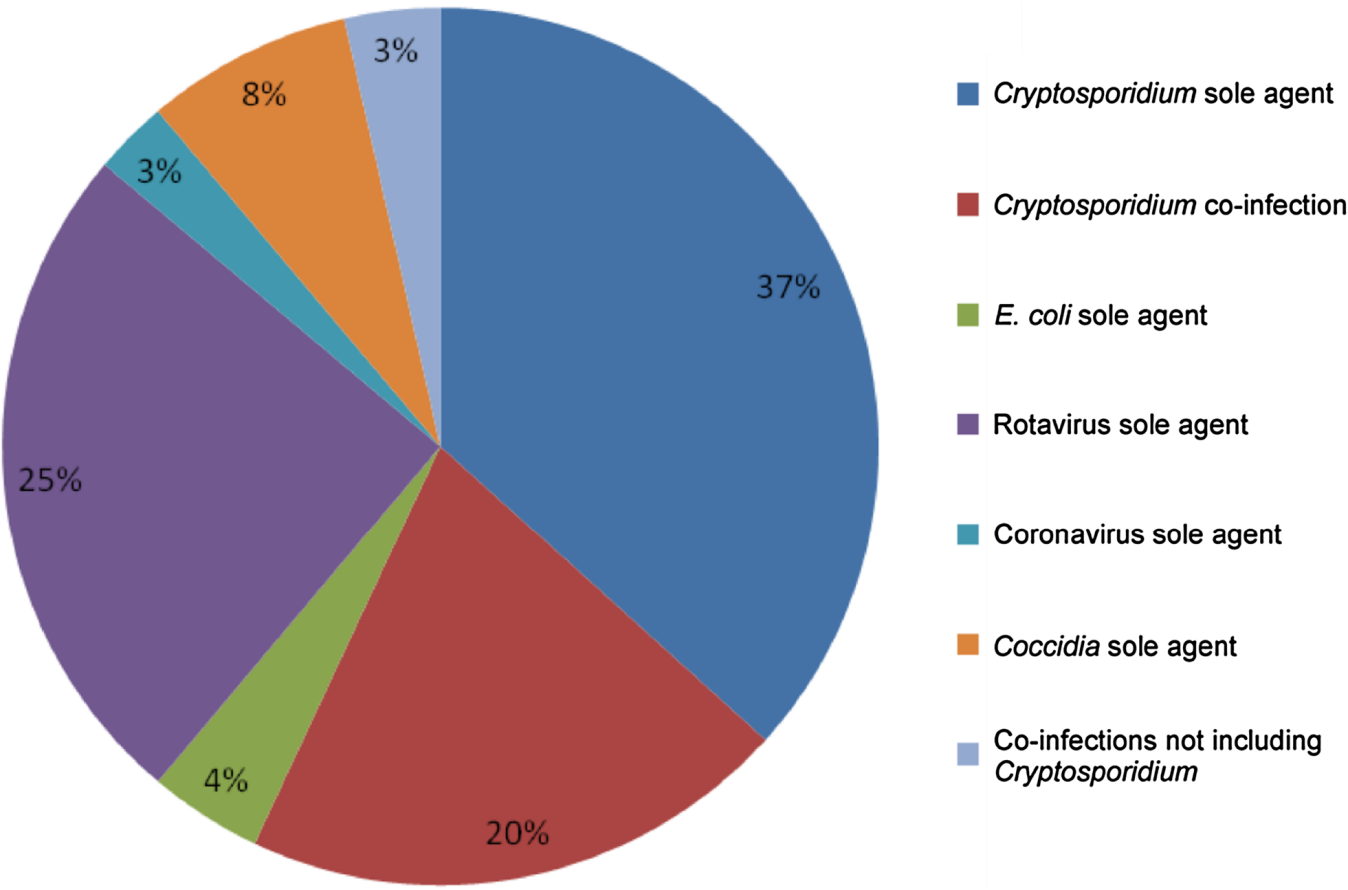
**Pathogens causing diarrhoea in young calves.** *Cryptosporidium* is the most commonly detected pathogen causing diarrhoea in calves less than 1 month of age as a proportion of diagnosable submissions 2007–2011 (veterinary investigation diagnosis analysis [VIDA]).

Cryptosporidiosis was first reported in cattle in the early 1970s [3], but the observed clinical disease could not be solely attributed to *Cryptosporidium* as there was evidence of co-infection with other viral and bacterial pathogens. In 1983, neonatal diarrhoea in experimentally infected calves was reported with *Cryptosporidium* species as the single infective agent [4]. Cryptosporidiosis is now recognised as endemic in cattle worldwide and is one of the most important causes of neonatal enteritis in calves globally [5–7]. Veterinary surveillance reports show cryptosporidiosis has been the main diagnosed cause of enteritis in calves in the UK between 2007 and 2011 (Figure 1) [8].

### Parasite life cycle

**Figure 2:**
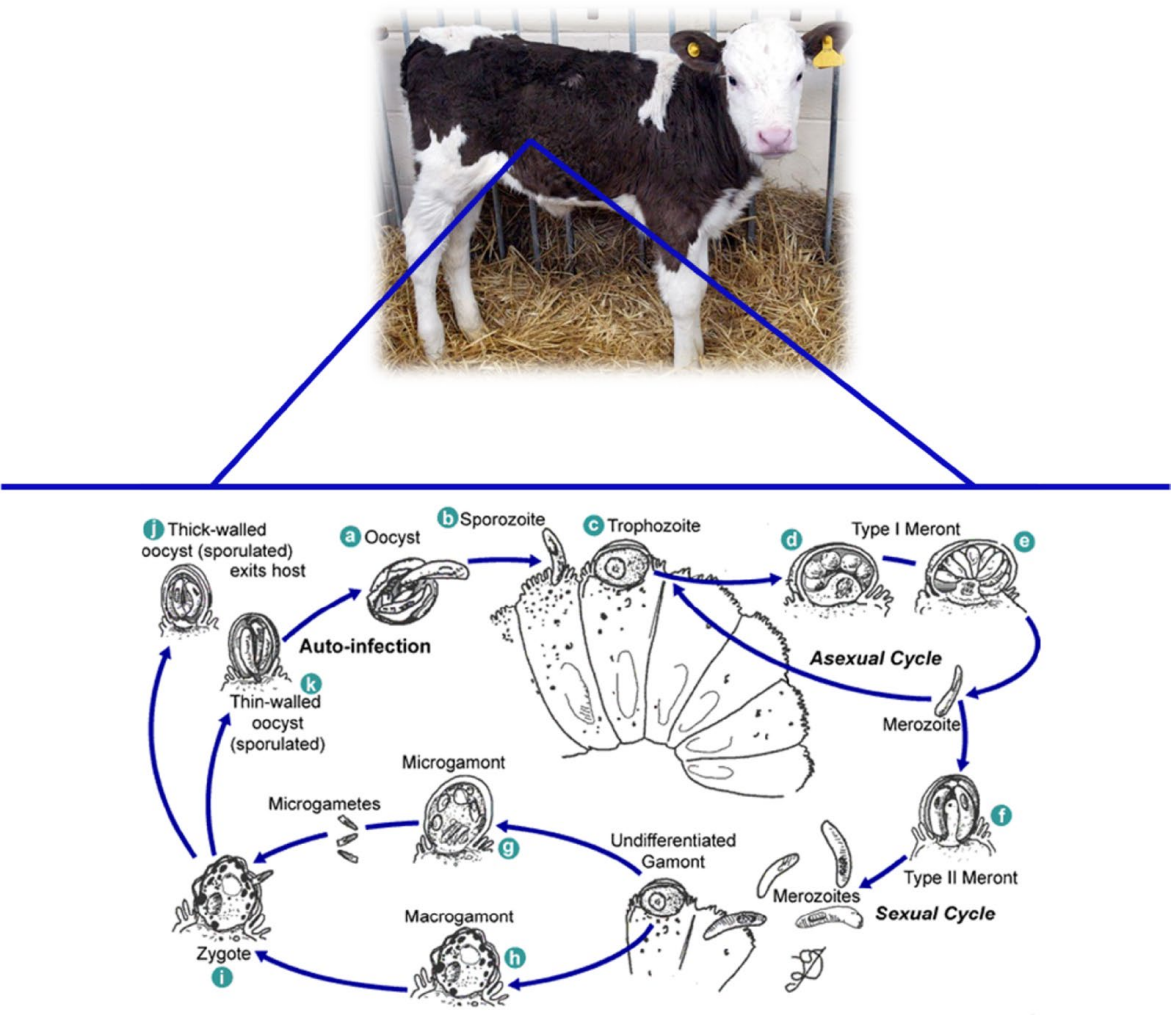
**Life cycle of** ***Cryptosporidium.*** Ingested sporulated oocysts release four sporozoites that invade host epithelial cells and develop into trophozoites, before undergoing asexual and sexual reproduction, resulting in the generation of both thin and think walled oocysts. Thin-walled oocysts auto-infect epithelial cells and thick-walled oocysts are excreted in the faeces of the host. Reproduced from [9].

**Figure 3:**
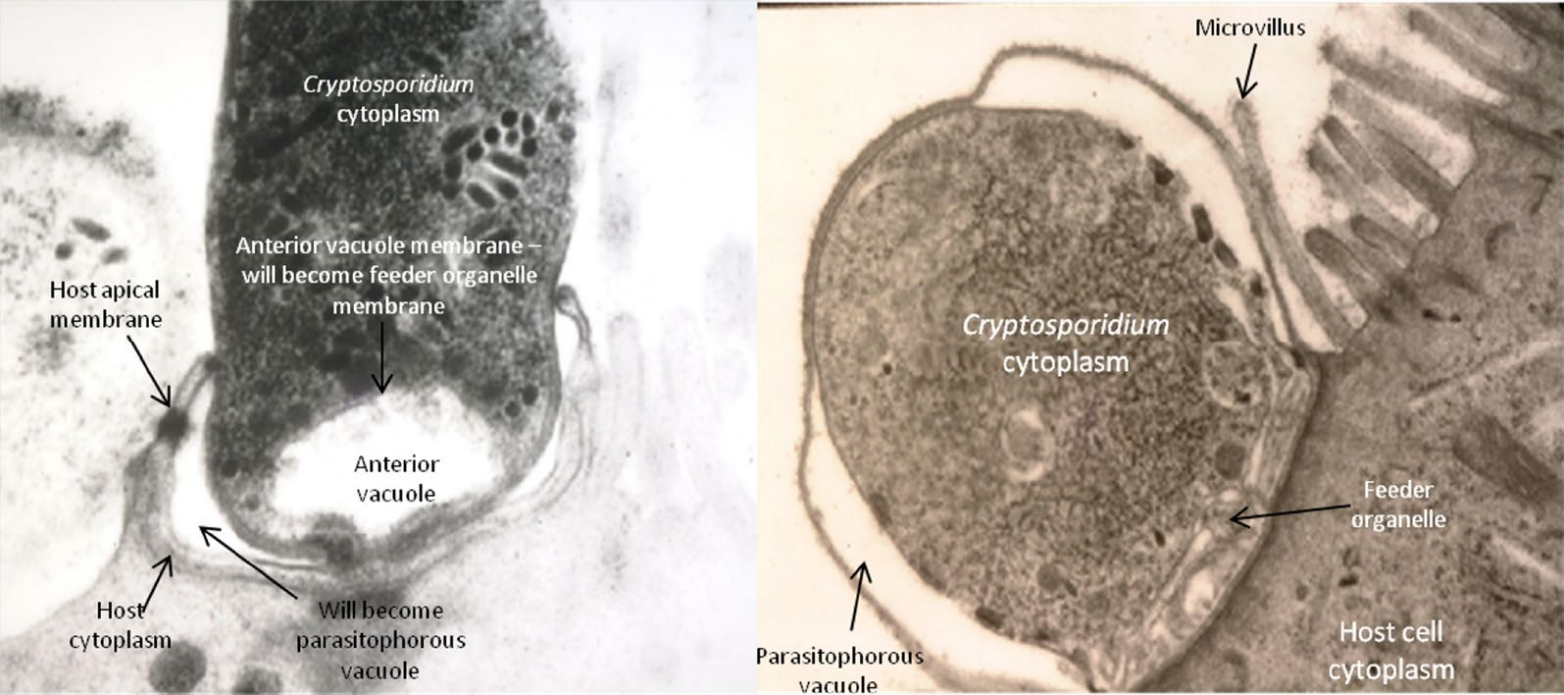
**Parasite invasion in host cells.** Images illustrate a *Cryptosporidium* sporozoite invading host epithelial cells (left) and a *Cryptosporidium* trophozoite within the parasitophorous vacuole (right). Images by kind permission of Saul Tzipori.

*Cryptosporidium* oocysts are transmitted between hosts via the faecal-oral route, either directly via contact with faeces from infected hosts, or indirectly through environmental contamination or ingestion of contaminated food or water. Following ingestion of infective *Cryptosporidium* oocysts by the host, the conditions in the gastrointestinal tract (low pH and body temperature) trigger oocyst excystation and four sporozoites are released (Figure 2A). *Cryptosporidium parvum* sporozoites adhere to epithelial cells (Figure 2B) of the ileum, specifically at the ileocaecal junction in the case of *C. parvum*. Following attachment, the sporozoites become incorporated within a parasitophorous vacuole formed by the host cell membrane yet remain extracytoplasmic. A feeder organelle, unique to *Cryptosporidium* and present in all intracellular stages, acts as the interface between the parasite and the host cell. The feeder organelle enables the parasite to obtain all necessary nutrients from the host while still being protected from the host immune response and hostile gut conditions (Figure 3).

After the development of the feeder organelle the sporozoite itself becomes more spherical in shape and forms a trophozoite (Figure 2C). The parasite begins asexual reproduction (Figure 2D) and develops into a type I meront (Figure 2E) which releases merozoites. The merozoites that are formed within the type I meront can immediately re-infect the host, by invading neighbouring epithelial cells and beginning asexual reproduction again, or develop into a type II meront.

Type II meronts release four merozoites that initiate the sexual reproductive cycle. The released merozoites invade host cells and differentiate into either macrogamonts (Figure 2H) or microgamonts (Figure 2G). Microgamonts develop multiple nuclei and release free microgametes that penetrate and fertilise the macrogamete, producing a zygote (Figure 2I). Meiosis occurs and the zygote differentiates into four sporozoites as the oocyst develops and is released from the lumen.

The sporozoites may be released directly into the lumen either from thin-walled oocysts (Figure 2K) that re-infect the host, or are contained in thick-walled oocysts (Figure 2J) which are shed in faeces and are immediately infective for other hosts [10]. The ability to produce thin-walled oocysts which enable auto-infection of the host is one of the reasons why the *Cryptosporidium* parasite is so successful. This auto-infection means that the parasite can produce many new oocysts in a relatively short time.

Thick-walled sporulated oocysts are excreted by infected hosts and are very resistant to many environmental conditions [11, 12]. Oocysts survive for several months in cool, moist climates, but can be inactivated by desiccation [13]. Environmental contamination and oocyst persistence is a significant factor in the epidemiology of bovine cryptosporidiosis.

## Bovine cryptosporidiosis

Four species of *Cryptosporidium* are commonly found in cattle: *C. parvum*, *C. bovis*, *C. ryanae* and *C. andersoni,* but only *C. parvum* is associated with clinical disease in neonatal calves [14, 15], with older animals (> 6 weeks) exhibiting asymptomatic shedding of oocysts.

There tends to be an age-related distribution [16] of these species with *C. parvum*, *C. bovis* and *C. ryanae* infecting the small intestine of weaned calves. *Cryptosporidium bovis* and *C. ryanae* have not yet been associated with clinical disease in any age group of cattle although one study from Sweden reported the presence of *C. bovis* as a single pathogen in diarrhoeal samples (n = 6) from calves aged > 21 days, this suggests that *C. bovis* may have pathogenic potential [17]. *Cryptosporidium andersoni* is more frequently found in adult cattle then younger animals and infects the abomasum [18]. The clinical signs associated with *C. andersoni* include a reduction in weight-gain and milk yield in adult cows [19].

One suggestion for the age-related distribution of *Cryptosporidium* species seen in several host species is that changes in the gut microflora as the animal matures or due to dietary changes may affect the ability of the parasite to infect the gut. While there are no experimental trials demonstrating this in cattle, Kvac et al. [21] investigated the age-related susceptibility of pigs to *C. scrofarum*; previous work had indicated that while *C. suis* infected all age groups of pigs it appeared that *C. scrofarum* only infected older animals [20]. In their study, groups of 4, 5, 6, 7 and 8 weeks-old naïve piglets were infected with *C. scrofarum* oocysts and faecal samples were collected and examined for the presence of oocysts. The study found that oocysts were only detected in the faeces of animals > 5 weeks of age. Additionally, no parasite DNA was detected in the gut samples of the 4 week old pigs at post-mortem while *C. scrofarum* DNA was detected in the duodenum, ileum and jejunum of the older pigs. The authors suggested that changes to the gut at weaning, while the piglets were adapting to a new diet may make the gut easier for the parasite to invade [21].

Neonatal animals infected with *C. parvum* may suffer from profuse watery diarrhoea, inappetence, lethargy, dehydration and in some cases death can occur. The onset of diarrhoea usually occurs around 3–4 days after ingestion of infective oocysts and lasts for approximately 1–2 weeks. Oocyst shedding occurs between 4 and 12 days post-infection though this can vary depending on the initial challenge dose [22] and oocyst shedding is not always associated with diarrhoea. Infected calves can shed large numbers (over 1 × 10^10^) of oocysts each day [23], which are immediately infective to other susceptible hosts [23]. Very few *C. parvum* oocysts are required to cause infection in susceptible hosts, although evidence suggests this varies according to parasite isolate. As few as nine oocysts of the TAMU isolate caused disease when tested on human volunteers, whereas in the same study the infective dose was 87 and 1042 oocysts for the IOWA and UCP isolates, respectively [24]. A similar study in neonatal calves (< 24 h old), experimentally infected with *C. parvum,* demonstrated that as few as 17 oocysts were sufficient to cause diarrhoea and oocyst shedding [25]. Since naturally infected calves can shed in excess of 3 × 10^10^ oocysts over a 6 day period [23], the ability of *C. parvum* to rapidly multiply in the gut and establish infection after exposure to a small number of oocysts makes cryptosporidiosis a difficult disease to control on farms.

### Prevalence

Bovine cryptosporidiosis is widespread and has been reported as a major cause of calf enteritis [5–7] in many countries throughout the world [26–29]. The prevalence of *C. parvum* infection varies between countries and studies. For example, reported *C. parvum* prevalence rates of *C. parvum* in pre-weaned calves in the UK range from 28.0 to 80.0% [28, 30, 31]. Studies from other parts of the world have reported prevalence of *C. parvum* in pre-weaned calves from 3.4 to 96.6% [16, 32–38]. A longitudinal study carried out on a single farm in the USA which followed a group of calves (n = 30) from birth to 24 months showed that 96.6% (29/30) of calves were positive for *Cryptosporidium* at 2 weeks of age [35]. This demonstrates the high likelihood that almost all calves on a farm become infected with *Cryptosporidium* in the first few weeks of life.

The variability in the reported prevalence of *C. parvum* most likely reflects differences in the design of the studies and the detection methods used. Animal age at the time of sampling is very important as calves aged < 6 weeks are most likely to be shedding *C. parvum* while older animals may shed another (non-pathogenic) *Cryptosporidium* species [14, 15]. The type of sample examined (diarrhoeic samples versus non-diarrhoeic), nature of the study (point-prevalence studies may underestimate prevalence) and the techniques employed to examine samples can have a significant impact on results. For example, one of the most common methods for veterinary diagnosis of cryptosporidiosis is microscopy. Though this one of the most cost-effective methods for detection of the parasite many microscopy methods require a degree of technical expertise and do not enable speciation of the parasite. Molecular tools are usually used in a research setting as these are much more sensitive and provide far more information about the parasite such as species and genotype [2]. Species can be differentiated using multiplex polymerase chain reaction (PCR) [39], restriction enzymes to digest PCR products into fragments of different sizes [PCR-restriction fragment length polymorphism (RFLP)] or by direct sequence analysis. Real-time PCR is can also be usedfor *Cryptosporidium* detection and can detect as few as 2 oocysts per PCR [40]. An advantage of real-time PCR is the ability to quantify parasite burden as standard PCR can only indicate the presence of parasite DNA but cannot quantify. During sample processing, a concentration step prior to microscopy or PCR can increase the likelihood of detecting the parasite [41, 42], particularly in large samples containing low oocyst numbers. It is important that the appropriate tools are utilised when investigating prevalence of *Cryptosporidium* as using less sensitive methods can lead to an underestimation.

### Economic and production impact

The precise economic losses associated with bovine cryptosporidiosis have not thus far been examined in detail but include the cost of treatment and management of enteritis, reduced feed conversion and production efficiency and losses due to animal death. Research by the Scottish Agricultural College, which examined 212 calf diarrhoea (not necessarily only *Cryptosporidium*) outbreaks investigated by 20 veterinary practices, estimated that the typical cost associated with management of disease was a minimum of £34 per calf affected, excluding labour [43]. Considering that in some cases it is possible that almost all calves within a herd may suffer from diarrhoea this cost is not insignificant.

At present, studies looking at the long term effects of *C. parvum* infection in calves have not been carried out. However, *Cryptosporidium* infection can impair growth rates in humans, lambs and mice [44–47]. A cohort study of children from a slum community in Southern India which were infected with *C. hominis, C. parvum* or *C. meleagridis* showed that children that had suffered from multiple bouts of cryptosporidiosis in the first 2 years of life were significantly lighter and shorter than children who had only a single episode at 2 years of age. However, by 3 years of age there were no significant differences in height and weight between children who had suffered from single or multiple episodes of cryptosporidiosis [44]. An earlier study carried out on children aged < 3 months in Peru also found that children infected with *C. parvum* were shorter and lighter than uninfected children. By 12 months after the onset of infection, children who had been infected were 1.05 cm shorter than their non-infected counterparts [45].

Very few studies have been carried out in livestock to determine the long term effects of cryptosporidiosis on growth. One study in Australia showed that lambs which were positive for *Cryptosporidium* were up to 1.65 kg lighter than uninfected lambs at slaughter [46]. A subsequent study involving > 1000 lambs from eight farms in Australia reported that shedding of *C. parvum* pre-slaughter was associated with a lower carcass weight of up to 2.6 kg compared to lambs which did not shed *C. parvum* [48]. If a similar reduction in the long term growth of calves affected by *Cryptosporidium* also occurs, this may prove costly for farmers due to loss of income from lower carcass weights, treatment cost or additional feed costs required to get calves to market weight.

### Zoonotic implications

Most human cases of cryptosporidiosis are caused by either the zoonotic species *C. parvum* or the human adapted species *C. hominis* [40]. Together these two species account for > 90% of human infections worldwide [49] and 96% of clinical cases in the UK [50].

Cryptosporidiosis in humans can be found worldwide in many environments, particularly in developing countries with poor sanitary conditions [51, 52]. Children (< 4 years of age) and the elderly are more susceptible to disease than young adults [53] with children tending to acquire the infection shortly after, or during weaning [54]. Although infection of immunocompetent people with *Cryptosporidium* tends to cause self-limiting diarrhoea, cryptosporidiosis is the second biggest cause (after rotavirus) of infant diarrhoea and death in Africa and Asia [55, 56]. In 2010, it was demonstrated that diarrhoea accounted for 10.5% of the 7.6 million deaths of children under the age of 5 [57]. Therefore, diarrhoea caused by *Cryptosporidium* can result in a significant number of deaths in developing countries.

In the UK, zoonotic transmission of *C. parvum* peaks in the spring months [58] and is thought to be related to springtime calving and lambing, and an increase in people participating in outdoor activities at this time of year. Cases of cryptosporidiosis decreased in the spring of 2001 during the foot and mouth disease outbreak, most likely due to a reduction in the number of young farm animals and restrictions on farm animal movement [59, 60]. Zoonotic transmissions to veterinary students working in practice for the first time are also common [61–64], in addition to outbreaks associated with petting zoos or farm visits [65, 66].

### Environmental impacts

The rapid amplification of *C. parvum* within infected hosts results in the production of significant numbers of oocysts. In addition, the inherent properties of oocysts that cause them to persist in the environment, means that oocysts represent a significant environmental threat to human health. Since one of the most common methods of transmission of *Cryptosporidium* is via contaminated water, maintaining water supplies *Cryptosporidium*-free is a major challenge for relevant government agencies and water companies. In the UK, livestock pasture often surrounds catchment areas collecting water ultimately destined for drinking water. Outbreaks of cryptosporidiosis in humans have often been attributed to contamination of water catchments by cattle manure. The hardy nature of *Cryptosporidium* oocysts and their small size makes it difficult to eliminate them from drinking water, and contamination to a water supply can potentially lead to large numbers of people becoming infected. Each year, 400–900 laboratory-confirmed cases of cryptosporidiosis are reported to Health Protection Scotland (HPS) and 3000–6000 cases in England and Wales are reported to the Health Protection Agency (HPA) [67]. In England and Wales between 2000 and 2003 there were six drinking water associated outbreaks of cryptosporidiosis, two of which were associated with *C. hominis* affecting 18 and 28 people, one with both *C. hominis* and *C. parvum* affecting 133 people. The remaining three outbreaks were all associated with *C. parvum* and affected 47, 3 and 4 people respectively [58].

A recent study which analysed the species and genotypes of *Cryptosporidium* present within livestock and wildlife grazing on a water catchment area with a history of cryptosporidiosis revealed that farm livestock (cattle and sheep) and wildlife (red and roe deer) all shed the same species of *Cryptosporidium* (*C. parvum*) which was detected in the local water supply [30]. Genotyping results showed that livestock and wildlife also shared the same genotype of *C. parvum* illustrating that transmission of the parasite can occur between livestock and wildlife where grazing is shared.

Having a broad range of hosts that can shed millions of oocysts, combined with that fact that a low dose of hardy oocysts can result in disease in naïve hosts make this parasite very widespread and difficult to manage.

## Current control measures for bovine cryptosporidiosis

Cryptosporidiosis is a difficult disease to control (due to environmentally stable oocysts, low infective dose and high levels of excreted sporulated oocysts) and infection may be transmitted to a group of susceptible hosts very quickly. The oocysts are resistant to many disinfectants [49, 68], there are no vaccines available to prevent the disease and available treatment options are limited and often rely on rehydration therapy [69].

### Farm management practices

As the oocysts of *C. parvum* are very difficult to eliminate from the environment an alternative control measure is to try and reduce the environmental contamination in the first place. Frequent removal of faeces and contaminated bedding from calving areas and calf houses, combined with steam-cleaning and disinfection with a suitable disinfectant such as Hydrogen Peroxide based disinfectants can help to reduce environmental build up. Thorough cleaning with very hot water followed by drying [70] can also be effective as the oocysts are susceptible to extremes of temperature (down to −20 °C and up to 60 °C) and desiccation [13].

### Therapeutics

At present, few products are licensed in the UK for the treatment or prevention of cryptosporidiosis in livestock or humans. Those which are available are not very effective, and in most cases will only reduce the duration of shedding and have little or no effect in immunocompromised patients.

#### Livestock

The only licensed treatment for cryptosporidiosis in calves is halofuginone lactate, the mechanism of action of this drug is unknown but it is thought to affect the merozoite and sporozoite stage of the parasite [71]. This drug is approved for use in both prevention and treatment of cryptosporidiosis in calves but cannot be used in animals have shown signs of diarrhoea for > 24 h. As a prophylactic measure the drug should be given within 48 h of birth and as a therapeutic agent, within 24 h of the onset of symptoms. Halofuginone lactate must be given for 7 consecutive days, which can be difficult to manage, particularly for beef calves that are kept with their dams. Treatment with halofuginone lactate does not completely prevent or cure disease but can reduce oocyst shedding and the duration of diarrhoea [72–74]. There are no licensed treatments for cryptosporidiosis in sheep, goats or pigs.

Several other chemotherapeutic agents have been tested for the treatment of cryptosporidiosis in livestock but none have resulted in a significant reduction in clinical symptoms. For example, some antibiotics, such as paromomycin have shown efficacy against *Cryptosporidium* oocyst shedding, clinical disease and mortality in calves, lambs and goat kids, but these compounds are not registered for use in calves [75, 76]. Fayer and Ellis [75] showed that experimentally infected calves treated with 100 mg/kg paromomycin twice daily for 11 days shed significantly less oocysts than control (untreated) calves. Treated calves also showed a significant reduction in duration and severity of diarrhoea. A field trial to test the efficacy of paromomycin in naturally infected dairy calves showed similar results; ten dairy calves on a farm known to have a problem with cryptosporidiosis were treated with 100 mg/kg paromomycin once per day for 10 days while another group were left untreated. Treated calves showed no diarrhoea until after treatment was stopped while untreated calves began to develop diarrhoea after 7 days. The authors suggested that the treated calves only showed diarrhoea after treatment was stopped due to the continuous parasite challenge in a field environment. Oocysts were detected in faecal samples from all calves but the shedding of oocysts in treated calves was significantly later than in untreated calves [77].

A few coccidiostats, such as decoquinate have been tested against *Cryptosporidium* in neonatal calves with limited or no reduction in oocyst shedding [78]. Nitazoxanide which is the only licensed treatment for human cryptosporidiosis has also been tested on experimentally infected calves and was shown to reduce duration of oocyst shedding and severity of diarrhoea in treated calves [79] but at present it is not licensed for use in cattle.

More recent studies which have evaluated novel bumped kinase inhibitors (BKIs) as a potential treatment for bovine cryptosporidiosis showed that experimentally infected calves treated with BKIs had a reduction in oocyst shedding when compared with untreated controls. One study did not show any differences in diarrhoea between the groups but the authors noted that the clinical disease seen in the control animals was very mild [80]. In another study it was demonstrated that all three of the BKIs that were tested alleviated clinical symptoms of cryptosporidiosis (when calves were dosed twice daily for 5 days) but did not eliminate them completely [81].

#### Vaccines

Currently, there are no vaccines available to prevent cryptosporidiosis in either farm livestock or humans. However, several attempts to develop such a vaccine have been made, some of which were partially successful under experimental conditions. Calves that were immunised with killed (γ-irradiated or lyophilised) *C. parvum* oocysts showed reduced oocyst shedding and diarrhoea when compared to non-immunised calves [82, 83]. However, this vaccine was not effective when tested under field conditions [84].

Infection with *Cryptosporidium* often occurs within the first week of life, so attempting to immunise the neonatal calves themselves is unlikely to be effective as this will not provide sufficient time to induce a significant immune response prior to infection [85]. To resolve this issue, attempts have been made to immunise pregnant cows to produce antibodies against *Cryptosporidium* which can be passed via colostrum to their calves. Calves receiving colostrum from cows vaccinated in this manner with recombinant *C. parvum* were protected against diarrhoea and also had reduced oocyst shedding, when compared to those calves that received colostrum from non-vaccinated cows [86]. However, a commercial vaccine has not so far become available as the efficacy of recombinant *C. parvum* has yet to be demonstrated in the field.

Despite several compounds being tested for efficacy against cryptosporidiosis in calves, to date, no new drugs have reached the market since 1999 when halofuginone lactate was approved for use. It is necessary to carry out further research to better understand this parasite so that new treatment options can be developed.

## Immunology of cryptosporidiosis

**Figure 4:**
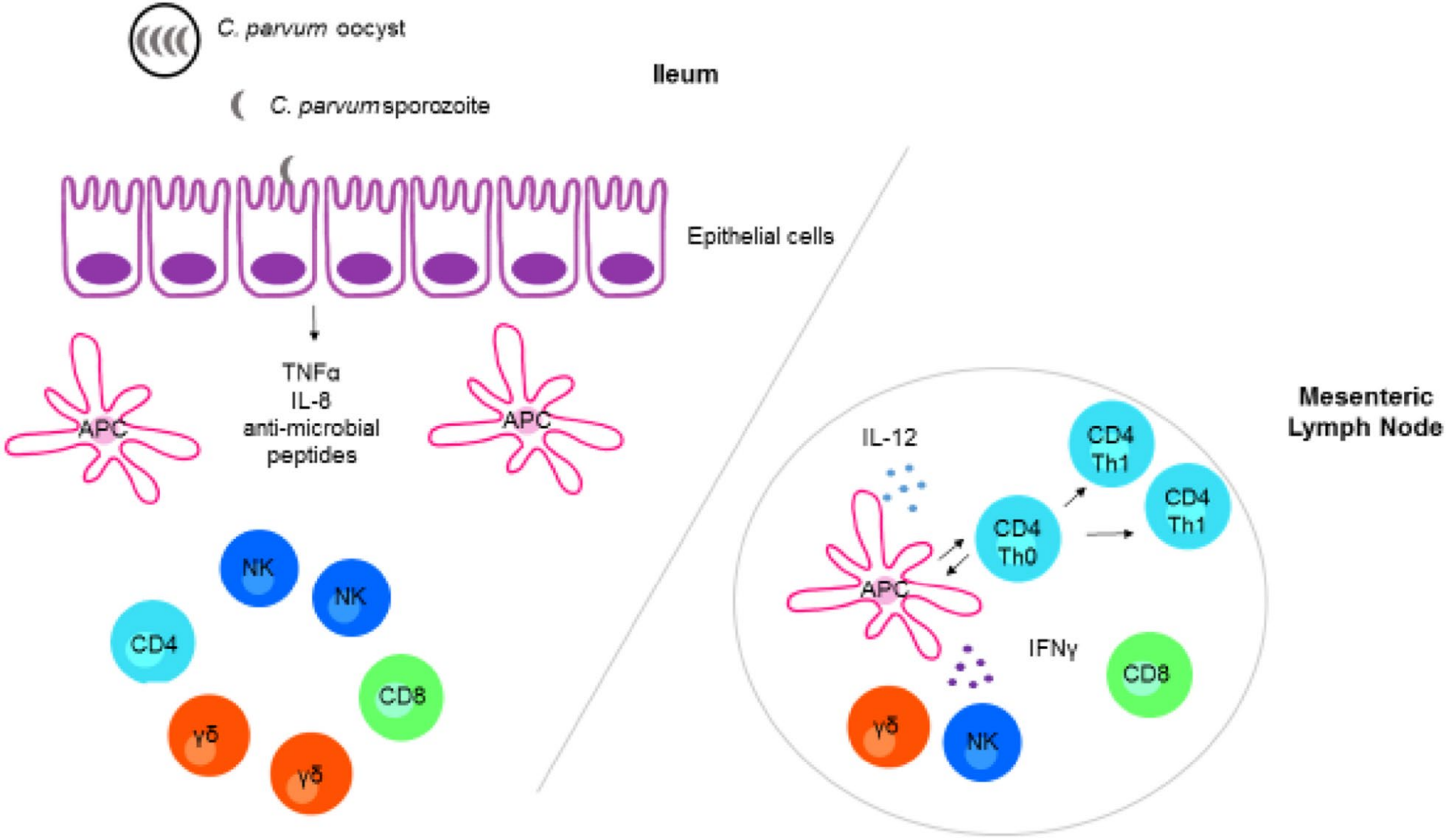
**Model of the immune response to** ***C. parvum.*** *C. parvum* oocysts are ingested by the host where they target the ileum of the small intestine and undergo excystation. Excysted sporozoites invade epithelial cells of the ileum where they complete asexual and sexual replication cycles. Infected epithelial cells produce pro-inflammatory cytokines, chemokines and anti-microbial peptides, which together orchestrate the immune response and recruit immune cell populations such as NK cells and γδ T cells to the site of infection. APCs such as DCs and macrophages sample antigen at the site of infection and following uptake, migrate to the draining mesenteric lymph nodes where they present antigen to CD4+ T cells. Antigen presentation together with the presence of IL-12 and IFNγ from APCs and NK cells/γδ T cells respectively, result in the generation of a Th1 CD4+ T cell response that is thought to be important during the immune response to *C. parvum* in humans, cattle and mice.

The development of future control methods against *Cryptosporidium* is likely to focus on vaccines and other immunotherapies. Therefore, there is an increased need to define the immunological parameters associated with disease progression. To date, most of the literature describing the immune response induced by *Cryptosporidium* parasites has been generated from studies in mice. Overall, the immune response to *Cryptosporidium* infection in humans and cattle is poorly understood. Studies in these clinically relevant hosts are urgently required in order to more effectively identify strategies to improve control of cryptosporidiosis. Here, we summarise our current understanding of the immune response to *C. parvum* infection (summarised in Figure 4).

### Innate immune response

#### Epithelial cells

Intestinal epithelial cells represent the primary cellular target for *Cryptosporidium* infection in vivo. Pathogen recognition receptors (PRRs) are involved in the detection of molecular features of pathogens by the innate immune system (reviewed in [87]). Among the different families of PRRs, the toll-like receptors (TLRs) have been shown to be important during *C. parvum* infection of intestinal epithelial cells. In cattle, mRNA levels of TLR2 and TLR4 were increased following infection of bovine intestinal epithelial cells with *C. parvum*, whereas the expression of TLR1, TLR3 and TLR5-TLR10 were unchanged. Interactions between *C. parvum* and TLR2/TLR4 expressed by bovine intestinal epithelial cells result in the activation of NFκB and subsequently, production of TNFα and IL-8 [88]. Similarly, infection of human biliary epithelial cells (cholangiocytes) with *C. parvum* induced recruitment of TLR2 and TLR4 to the attachment site of infected epithelial cells [89] and infection of the human epithelial cell line HCT-8 with *C. parvum* has also been shown to induce the expression of the pro-inflammatory cytokine IL-18 [90]. In addition to production of pro-inflammatory cytokines and chemokines, bovine and human intestinal epithelial cells secrete antimicrobial peptides after invasion by *Cryptosporidium*. For example, mRNA levels of β-defensin were increased in the intestine of *C. parvum*-infected calves compared with control tissues [91]. The human epithelial cell lines HCT-8 and HT-29 secrete increased levels of LL-37 and α-defensin-2 respectively after IL-18 stimulation, which could be a mechanism adopted by the host to limit *Cryptosporidium* [90]. As mentioned previously, *C. parvum* infection of human cholangiocytes attracted TLR2 and TLR4 to the cell surface, and inhibition of this TLR signalling resulted in their reduced expression of β-defensin 2 [89]. Taken together, these data suggest that TLR signalling through bovine and human epithelial cells during *C. parvum* infection results in the production of pro-inflammatory cytokines, chemokines and antimicrobial peptides. Whether these responses are important to induce a protective immune response to infection remains to be determined.

#### Natural killer (NK) cells

Natural killer cells (NK) are large granular lymphocytes, which were identified in the 1970s by their ability to lyse malignant or transformed cells without prior sensitisation [92]. This heterogeneous cell population has diverse roles in the immune system and NK cells are the first line of defence in the control of viruses, as well as certain intracellular bacteria and parasites [93–96]. Upon activation by the cytokines TNFα, IFNα/IFNβ, IL-12, IL-15 or IL-18 produced by accessory cell populations such as antigen presenting cells (APC), NK cells have two main effector functions. Firstly, NK cells are a significant source of immunoregulatory cytokines, primarily IFNγ but also TNFα, GM-CSF [97–99], IL-10 [98, 99] and IL-22 [100, 101]. Secondly, NK cells display cytotoxicity to target cells through the release of preformed, cytotoxic granules containing perforin and granulysin [102]. NK cells comprise 10–15% of peripheral blood lymphocytes in humans [102]. Within bovine peripheral blood, NK cells represent 0.5–10% of the total lymphocyte population with an increased prevalence in neonatal calves, particularly those aged between 8–120 days old. In parallel with humans and mice, the frequencies of circulating NK cells in bovine blood decline with age [103–105].

Natural killer cells exist as distinct subsets across species, for example, human NK cells are broadly subdivided based on their differential expression of the cell surface markers CD56 and CD16 (FcγRIII) [106, 107]. CD56 is not expressed in mice, therefore murine NK cells are distinguished by the expression of NK1.1 or CD49b [108] and can be further subdivided based on CD27 and CD11b expression [109]. NKp46 (NCR1; CD335), a natural cytotoxicity receptor expressed exclusively by NK cells, has been used to identify bovine, ovine and porcine NK cells and is recognised as a pan-species marker of NK cells [110–113].

The role of NK cells during *C. parvum* infection has been studied in ovine, murine and human models of cryptosporidiosis. Within the first few days following infection of 1 day old lambs with *C. parvum*, the abundance of NKp46+ NK cells expressing CD16, CD25 and perforin was increased in the gut [114]. In mice, it was demonstrated that oocyst shedding was higher in *C. parvum*-infected Rag 2^−/−^ γ_c_^−/−^ mice (mice lacking B, T and NK cells) compared with *C. parvum*-infected Rag2^−/−^ mice (mice lacking B and T cells). Rag 2^−/−^ γ_c_^−/−^ mice developed morbidity and died after *C. parvum* infection, but Rag2^−/−^ mice remained healthy, thus highlighting a role for NK cells in reducing disease severity during murine *C. parvum* infection [115]. Although NK cells are an important source of IFNγ, Rag2^−/−^ γ_c_^−/−^ mice were able to produce IFNγ despite their lack of NK cells, suggesting an alternative source of this cytokine during *C. parvum* infection [115].

Natural killer cells may also play a role in the clearance of *C. parvum* as human CD3− CD16+ CD56+ NK cells activated by IL-15 were able to lyse *C. parvum*-infected epithelial cells in vitro. Lysis of epithelial cells was mediated by engagement of the NKG2D receptor expressed by NK cells, with ligands major histocompatibility complex (MHC) class I chain-related protein A (MICA) and B (MICB) on epithelial cells [116]. Therefore, NK cells may play a role in the early protective immune response to *C. parvum* infection in sheep, mice and humans, but their role in bovine cryptosporidiosis remains to be elucidated.

#### γδ T cells

γδ T cells, which have a unique T cell receptor (TCR) consisting of one γ chain and one δ chain, constitute a minor fraction (5–10%) of the circulating lymphocyte population in humans and mice, but are more abundant in the gut mucosa. However, γδ T cells are a major subset of lymphocytes in ruminants and constitute up to 60% of circulating T cells in calves less than 6 months of age [117]. γδ T cells are involved in antigen presentation, cytokine production and regulation of the immune response.

Following *C. parvum* infection of calves, there was a significant increase in the frequency of γδ T cells in the ileum at day 3 post infection [118]. In a study comparing *C. parvum* infected-TCRα or TCRδ deficient mice, which lack αβ T cells and γδ T cells respectively, it was demonstrated that TCRα deficient mice were unable to clear the parasite whereas TCRδ deficient mice were able to clear *C. parvum*, albeit at a slower rate than wild type (control) mice. The authors concluded that γδ T cells are involved in the initial control of *C. parvum* infection in mice and αβ T cells are necessary for complete clearance of the parasite [119]. Thus, evidence suggests that γδ T cells may be involved in the early protective immune response to *C. parvum* in cattle and mice.

#### Dendritic cells (DCs)

Dendritic cells (DCs) are a heterogeneous population of potent APC that are essential mediators of immunity [120] and tolerance [121]. Stem cell precursors in the bone marrow give rise to circulating myeloid or lymphoid precursors that enter tissues and reside as immature DCs. Monocytes, macrophages and DCs share a common progenitor [122]. The cytokine Flt3L drives differentiation of these progenitor cells into DCs [123] and loss of Flt3L [124], Flt3 [125] or downstream signalling molecule STAT3 [126] reduces the number of DCs in vivo.

Dendritic cells are predominantly found underlying body surfaces including the skin, intestine and the trachea. They act as sentinels and respond to infection, inflammatory signals or tissue damage by migrating away from the periphery towards draining lymph nodes where they present antigen and initiate primary T cell mediated immune responses [127]. Two weeks after oral exposure of mice to *C. parvum*, live parasites were detected in the mesenteric lymph nodes and were associated with CD11c + DCs therefore suggesting DCs could take up *C. parvum* in vivo and migrate to the local lymph nodes [128]. During migration, DCs undergo maturation that is characterised by reduced endocytosis and an augmented expression of MHC molecules, costimulatory molecules and adhesion molecules. Following in vitro infection of mouse bone marrow-derived dendritic cells (BMDCs) with *C. parvum* sporozoites, DCs increased their expression of CD40, CD80 and CD86. In addition to augmented expression of these co-stimulatory molecules, BMDCs exposed to *C. parvum* produced pro-inflammatory cytokines including TNFα, IL-6 and IL-12 [128]. Production of IL-12 by *C. parvum*-infected BMDCs from C3H/HeJ mice (which lack a functional TLR4 pathway) was defective, suggesting that TLR4 signalling is important for production of IL-12 by *C. parvum*-infected BMDCs [128].

Interestingly, neonatal mice have a reduced number of intestinal CD103+ DCs due to a weak production of chemokines (CCL3, CCL4, CCL5, CCL22, CXCL9 and CXCL10) by intestinal epithelial cells, which results in an increased susceptibility to *C. parvum* infection. Injecting neonatal mice with Flt3L in vivo increased the number of CD103+ DCs and coincided with their improved resistance to infection [129]. Conversely, CD11c-DTR-Tg mice (depleted of CD11c+ DCs) have an increased susceptibility to infection with *C. parvum.* Adoptive transfer of DCs stimulated with live sporozoites reduced the parasite load therefore illustrating the importance of DCs as mediators of *C. parvum* infection in mice [130].

#### Macrophages

Macrophages are a diverse population of specialized phagocytic cells that are essential for host defense, homeostasis and wound repair. They are derived from bone marrow precursors and circulating blood monocytes, which differentiate into resident macrophages or DCs upon tissue entry [131]. Similar to DCs, the functions of macrophages include phagocytosis (they are known as the ‘professional phagocytes’), antigen presentation and cytokine production.

Rag2^−/−^ γ_c_^−/−^ mice are able to produce IFNγ despite their lack of NK cells, which suggested an alternative source of innate IFNγ during *C. parvum* infection [115]. Further extension of this work demonstrated that depletion of macrophages, by treatment with clodronate-containing liposomes, reduced resistance to *C. parvum* infection in mice. Importantly, mice depleted of macrophages were unable to produce IFNγ, indicating that macrophages were the alternative source of IFNγ during *C. parvum* infection. In addition, the authors showed that IL-18 was an important factor for immunity to *C. parvum* in Rag2^−/−^ γ_c_^−/−^ mice and IL-18 promoted production of IFNγ by macrophages during infection [132]. The inability of IFNγ gene-knockout mice to control *C. parvum* infection was associated with reduced recruitment of macrophages and T cells to the lamina propria following infection suggesting they play an important role in control of infection in mice [133]. The role of macrophages in human and ruminant models of cryptosporidiosis is currently unknown.

### Adaptive immune response

#### CD4+ T cells

Cell mediated immunity, particularly CD4+ T cells, is essential for the protective immune response to *Cryptosporidium* infection. This is highlighted by the increased susceptibility of acquired immune deficiency syndrome (AIDS) patients (who have low numbers of CD4+ T cells) to *C. parvum* infection [134], and ability of the parasite to cause chronic infections in MHC class II deficient mice (which lack functional CD4+ T cells) [135]. Importantly, cryptosporidiosis can be resolved in AIDS patients following restoration of their CD4+ T cell levels [135].

#### Th1 immune responses

A major function of CD4+ T cells is to act as helper lymphocytes, and upon interaction with antigen presenting cells, naïve CD4+ T cells can differentiate into various helper cell subsets. Differentiation of naïve CD4+ T cells, is dictated in part, by the cytokine milieu present at the time of differentiation. For example, the presence of IL-12 and IFNγ in the local environment results in the development of a Th1 immune response which is characterised by the production of TNFα, IL-2 and IFNγ [136]. Th1 biased immune responses are important for protection against intracellular pathogens and are involved during *Cryptosporidium* infection. In bovine studies, stimulation of PBMCs from *C. parvum*-infected calves with antigen derived from *C. parvum* oocysts resulted in an increased production of IFNγ by CD4+ T cells [137]. PBMCs from neonatal calves experimentally infected with *C. parvum* had increased mRNA levels of the Th1 cytokines from day 3 post-infection [138]. Similarly, expression of IL-12p40 and IFNγ by lamina propria and intraepithelial lymphocytes were noted following *C. parvum* infection of neonatal calves [139]. Similarly, a case of severe cryptosporidiosis was reported in a patient with an IFNγ deficiency [140] and IFNγ gene-knockout mice are highly susceptible to infection [141]. Treatment of immunocompetent or immunodeficient mice with exogenous IL-12 prior to infection with *C. parvum* reduced the severity of infection in an IFNγ dependent manner [142]. However, in a study comparing Rag2^−/−^ and wild type neonatal mice, it was demonstrated that CD4+ T cells were not required for control of *C. parvum* infection [143].

Taken together, these studies highlight the importance of Th1 polarised CD4+ T cell responses during *C. parvum* infection of cattle, humans and mice.

#### Th2 immune responses

Th2 polarisation of the immune response is driven by the presence of an IL-4 rich cytokine milieu which induces the differentiation of naïve CD4+ T cells into the Th2 subset of CD4+ T cells, characterised by their production of IL-4, IL-5, IL-10 and IL-13. Th2 responses are important in defence against extracellular pathogens, allergy/asthma and antibody mediated immunity [144].

Treatment of C57BL/6 mice with anti-IL-4 monoclonal antibody prior to infection with *C. parvum* caused prolonged excretion of *C. parvum* oocysts for at least 11 days longer than control mice. Furthermore, IL-4 deficient mice also exhibited prolonged excretion of *C. parvum* oocysts, which lasted 23 days longer than mice with intact IL-4 [145]. Interestingly, in a study using IL-4 deficient mice, IL-4Rα deficient mice and IL-4 neutralising antibody, it was demonstrated that production of IL-4 during *C. parvum* infection of BALB/c mice induced expression of IFNγ and IL-12 and therefore promoted Th1 immunity in the intestine [146]. Thus, production of the Th2 cytokine IL-4 in response to murine *C. parvum* infection may drive protective Th1 immune responses.

#### Th17 immune responses

A third subset of CD4+ T cells, distinct from Th1 and Th2 CD4+ T cells, was described in 2005 and named the Th17 subset of CD4+ T cells [147]. The presence of TGFβ, IL-6 and IL-23 in the local environment results in differentiation of Th17 cells which are characterised by the production of IL-17 (also known as IL-17A). Th17 CD4+ T cells are important in host defence against a wide range of pathogens, for example, through recruitment of macrophages and neutrophils to the site of infection [148]. In mice immunosuppressed by dexamethasone and then infected with *C. parvum*, it was shown that mRNA levels of TGF-β and IL-6 in the gut associated lymphoid tissue (GALT) increased at 6 and 12 h post-infection respectively. This was alongside increased levels of STAT-3 and the transcription factor RORγT, which promote differentiation of naïve CD4+ T cells into Th17 cells. Consequently, levels of IL-17 protein increased in the GALT after infection [149]. There is little known about the possible role of Th17 immune response during the bovine immune response to *C. parvum* infection.

#### CD8+ T cells

CD8+ T cells, alongside CD4+ T cells and γδ T cells, are present at an increased frequency within the ileum of *C. parvum*-infected calves at day 3 post-infection, compared with tissues from uninfected calves [118]. Furthermore, following infection of 1 day old lambs with *C. parvum*, CD8+/NKp46− lymphocytes (which the authors concluded were most likely to be CD8+ T cells) were significantly increased in the small intestine at day 3 and day 6 post infection, reflecting their recruitment following infection [114]. Therefore, CD8+ T cells appear to be involved in the immune response after infection of calves and lambs with *C. parvum.* Conversely, in studies using MHC class I deficient mice, which lack CD8+ T cells, it was shown that mice were not susceptible to *C. parvum* infection whereas mice which lacked CD4+ T cells due to a MHC class II deficiency were highly susceptible to infection. Thus it appears that CD4+ T cells, rather than CD8+ T cells, are important during murine *C. parvum* infection [150].

#### B cells

The relevance of B cells and immunoglobulins during *Cryptosporidium* infection remains controversial. Mice depleted of B cells (and therefore unable to produce appropriate levels of protective antibody) were able to control *C. parvum* infection to a similar level as mice with intact B cells [151]. Using gene-targeted B cell deficient mice, it has also been shown that B cells were not required for resistance to initial *C. parvum* infection or resolution of infection [152]. Data from these studies suggest that B cells and antibody-mediated immune responses may not be necessary for protection against murine *C. parvum* infection. Whether the same is true for protection against *Cryptosporidium* infection in natural host species remains to be determined.

#### Models to study the interaction between *Cryptosporidium* and the host

Generally, mice infected with *C. parvum* have limitations when used as models for *Cryptosporidium* infection in humans or cattle. Additionally, bovine-specific examples such as the significantly increased frequency of nonconventional T cells, ultralong complementarity-determining region 3 (CDR3) domain antibodies and uniquely expanded NK receptor repertoires [153–155] illustrate the many fundamental differences between the murine and bovine immune systems. The impact that these factors may play during *Cryptosporidium* infection cannot be captured using mouse models. Improved in vitro models would also ideally be developed in order to enable the study of the interaction between *C. parvum* and the host. To date, epithelial cell lines (particularly the HCT-8 cell line) have been utilised to decipher the interaction between the parasite and human intestinal epithelial cells. Whilst these studies are valuable, epithelial cell lines are not fully representative of the complex structure and cellular diversity of the intestinal epithelium, which consists of many cell types including epithelial cells, goblet cells, stem cells, Paneth cells and enteroendocrine cells. Intestinal organoids, or ‘mini guts’, appear to represent an example of a physiologically-relevant in vitro system that has the potential to fill this important requirement gap. These are three dimensional structures, differentiated in vitro from intestinal stem cells, which recapitulate the structure of the intestinal epithelium in vivo and contain all of the cell types associated with the intestine. Organoids would be excellent tools to study the early interactions of *Cryptosporidium* with the gut epithelium, as pathogens can be introduced into the lumen of the intestinal organoids in vitro enabling their relationship with the host to be deciphered in a physiologically relevant context. Zhang et al. provided the first study of *C. parvum* and intestinal organoids, which were derived from mouse tissue. The authors illustrated that the presence of *C. parvum* attenuated the differentiation of murine intestinal organoids [156]. Therefore, similar in vitro systems would be advantageous to understand the nature of the host–pathogen interaction during *C. parvum* infection in cattle, and other relevant host species.

## Concluding remarks

Despite being discovered over 100 years ago *Cryptosporidium* remains one of the most difficult pathogens to control on farms and in the environment. To date, progress in the development of effective treatments for humans or animals has been slow. This may be due, in part, to the lack of understanding of the cellular and molecular interactions that occur in the gut mucosa during the early stage interactions between host and parasite. The majority of studies that have been carried out have focussed on mice, an asymptomatic model. Therefore, a more in-depth understanding of the pathogenesis of *Cryptosporidium* infection in clinically relevant hosts is urgently required. Data derived from these studies is necessary to help identify the factors that influence disease resistance and recovery, and to aid the development of effective control strategies to help control this important disease.

## Abbreviations

%: percent
~: approximately
<: less than
=: equal to
>: more than
°C: degrees centigrade
AIDS: acquired immune deficiency syndrome
APC: antigen presenting cell
BMDCs: bone marrow-derived dendritic cells
*C. andersoni*: *Cryptosporidium andersoni*
*C. bovis*: *Cryptosporidium bovis*
*C. hominis*: *Cryptosporidium hominis*
*C. meleagridis*: *Cryptosporidium meleagridis*
*C. parvum*: *Cryptosporidium parvum*
*C. ryanae*: *Cryptosporidium ryanae*
CD: cluster of differentiation
CDR: complementarity-determining region
cm: centimetre
DCs: dendritic cells
DNA: deoxyriboneucleic acid
GALT: gut associated lymphoid tissue
GM-CSF: granulocyte-macrophage colony-stimulating factor
HCT-8: human colon carcinoma cell line
HPA: health protection agency
HPS: health protection Scotland
HT-29: human colon adenocarcinoma cell line
ID50: infectious dose to 50% of exposed individuals
IFN: interferon
IL: interleukin
kg: kilogram
MHC: major histocompatibility complex
MICA: MHC class I chain-related protein A
MICB: MHC class I chain-related protein B
mRNA: messenger RNA
NFκB: nuclear factor kappa-light-chain-enhancer of activated B cells
NK: natural killer cells
NKG2D: natural killer group 2 member D
PBMCs: peripheral blood mononuclear cells
PCR: polymerase chain reaction
pH: negative log of the activity of the hydrogen ion in an aqueous solution
PRRs: pathogen recognition receptors
Rag2: recombination activating gene 2
RFLP: restriction fragment length polymorphism
RORγt: retinoid-related orphan receptor gamma t
spp.: species
STAT3: signal transducer and activator of transcription 3
TCR: T cell receptor
TGF: transforming growth factor
Th: T helper
TLR: toll-like receptors
TNF: tumor necrosis factor
UK: United Kingdom
USA: United States of America
VIDA: veterinary investigation diagnosis analysis
α: alpha
β: beta
γ: gamma
δ: delta
